# New alleles of the SWI/SNF chromatin remodeling complex gene *phf-10*

**DOI:** 10.17912/micropub.biology.000533

**Published:** 2022-03-17

**Authors:** Laura D Mathies, GinaMari Blackwell, Jill C Bettinger

**Affiliations:** 1 Department of Pharmacology and Toxicology, Virginia Commonwealth University, Richmond, VA 23298, USA

## Abstract

SWI/SNF chromatin remodeling complexes regulate many aspects of metazoan development and mutations in SWI/SNF genes are associated with diverse human diseases including cancer and alcohol use disorder. In *C. elegans,* SWI/SNF subunits are required for viability, somatic gonad development, and normal behavioral responses to ethanol. SWI/SNF complexes can be classified as BAF (BRG1/Brm-associated factors) or PBAF (Polybromo-associated BAF) based on their subunit composition. While there are loss-of-function alleles for most SWI/SNF family members, strong loss of function mutations have not previously been reported for the PBAF gene *phf-10.* Here we describe two new alleles of *phf-10* that we generated using CRISPR/Cas9 genome editing.

**Figure 1. New alleles of phf-10 affect development and behavioral responses to ethanol.**
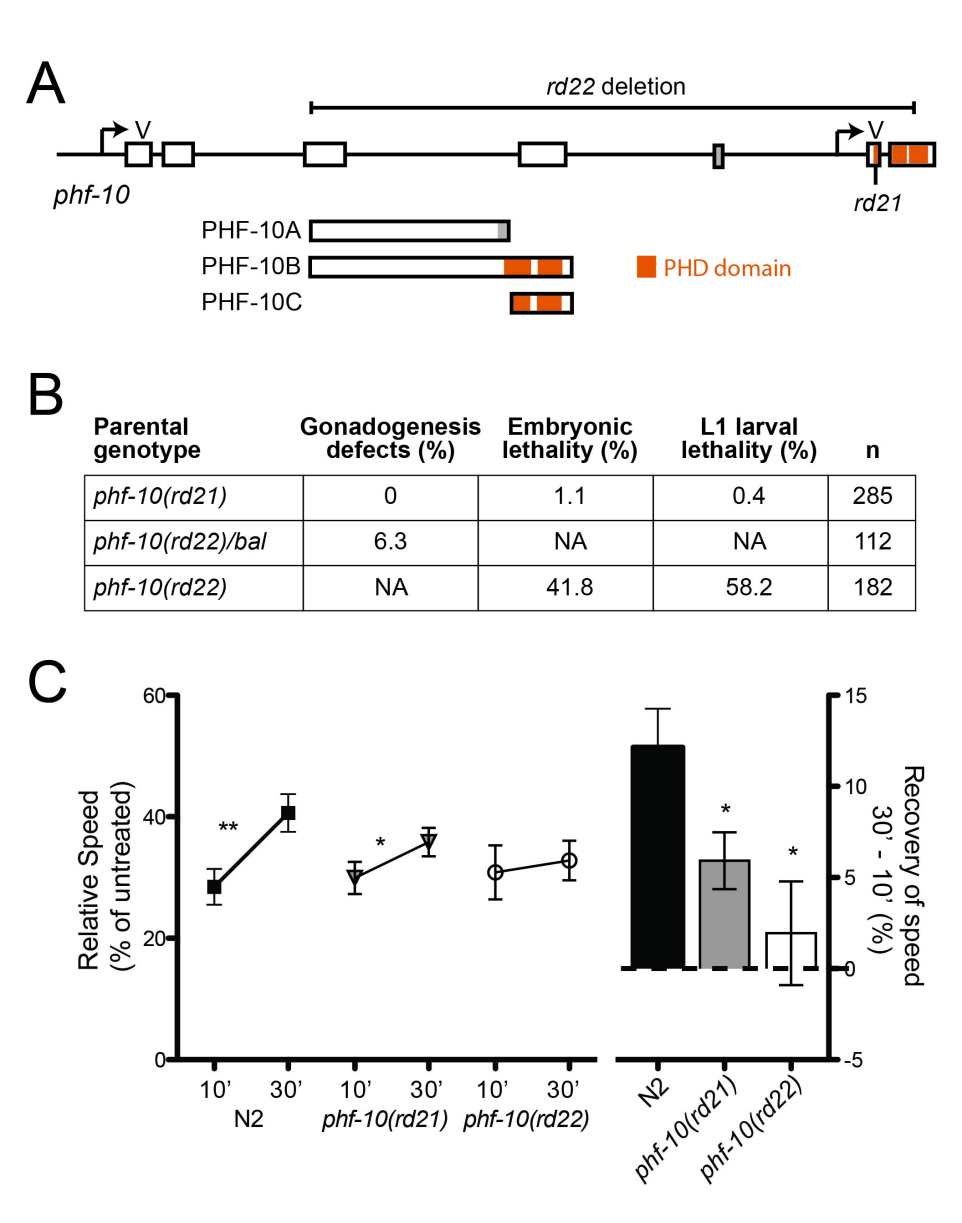
A. The *phf-10* locus produces at least three isoforms from two promoters. An alternatively spliced exon is indicated in grey and PHD domains are indicated by orange bars. Two new alleles of *phf-10* , *rd21* and *rd22* , were created by CRISPR/Cas9 genome editing using guide RNAs targeting the first and fifth exons; V indicates cut sites. B. Effects of *phf-10* alleles on development. Homozygous *phf-10* mutants were derived from the indicated parental genotype; *n* = number of homozygous mutant animals scored. *phf* - *10(rd22)* mutants had a low percentage of zygotic gonadogenesis defects and completely penetrant maternal effect embryonic and larval lethality. *phf-10(rd21)* mutants had very little lethality and no gonadogenesis defects. NA, not assessed. C. Acute behavioral responses to alcohol. Wild type worms (N2) develop AFT to ethanol, as indicated by a statistically significant increase in relative speed between 10 and 30 minutes of exposure. The degree of AFT is quantitated on the right Y-axis. Two loss-of-function alleles of *phf-10* have reduced AFT to ethanol. *phf-10(rd21)* mutants develop AFT (left), but the degree of AFT is significantly less than N2 (right). *phf-10(rd22)* mutants do not develop AFT. Paired two-tailed Student’s t tests were used for statistical comparisons; **p ≤ 0.01, *p ≤ 0.05.

## Description

We generated new loss-of-function mutations in *phf-10* using CRISPR/Cas9 genome editing. There are three described isoforms of PHF-10, resulting from multiple promoters and alternative splicing (Fig. 1A). In order to identify potentially null alleles, we used two guide RNAs designed to target the 5’ and 3’ ends of the gene and looked for large deletion events. We identified two alleles, *rd21* and *rd22* (Fig. 1A). The *rd22* allele is a 4119 bp in-frame deletion that is predicted to result in a PHF-10B protein lacking amino acids 149 to 387, including the first PHD domain and part of the second PHD domain; PHF-10C is predicted to be absent and PHF-10A is predicted to be truncated after amino acid 148. We identified the *rd21* allele based on its gonadal phenotype; *rd21* is a 4 bp deletion resulting in a frame shift of PHF-10B after amino acid 329; it is predicted to have no effect on PHF-10A or PHF-10C (the mutation is upstream of the start of PHF-10C translation). The *rd21* and *rd22* alleles are expected to have different effects on *phf-10* function as a result of their different molecular effects on the three PHF-10 isoforms.

*C. elegans* SWI/SNF chromatin remodeling complexes are required for a wide range of developmental processes, and loss-of-function alleles of SWI/SNF genes frequently result in embryonic or larval lethality (Cui et al. 2004; Large and Mathies 2014; Sawa et al. 2000; Shibata et al. 2012; Weinberg et al. 2013). We examined the *phf-10* alleles for effects on viability and found that both alleles are homozygous viable; *rd21* can be maintained as a homozygote, while *rd22* is maternal effect lethal with its progeny dying as embryos or L1 larvae (Fig. 1B). SWI/SNF complexes control multiple aspects of somatic gonad development and loss-of-function mutations result in missing gonadal arms (Large and Mathies 2014; Shibata et al. 2012). We examined the *phf-10* alleles for effects on somatic gonad development and observed a low penetrance phenotype of missing gonadal arms in *phf-10(rd22)* (Fig. 1B), indicating that *phf-10* plays a role in gonadogenesis. We did not observe gonad defects in *phf-10(rd21).* Together with our molecular analysis, these results suggest that *phf-10(rd22)* is a strong loss-of-function and possible null allele and *phf-10(rd21)* is a weak hypomorphic allele.

SWI/SNF subunits are also required for normal behavioral responses to alcohol (Mathies et al. 2015). We used locomotion assays to assess the behavioral responses to ethanol of the new *phf-10* alleles (Fig. 1C). Wild-type worms have significantly reduced locomotion speed after a 10-minute exposure to ethanol (a measure of initial sensitivity) and they develop acute functional tolerance (AFT) to ethanol after 30 minutes, as indicated by an increase in locomotion speed at 30 minutes relative to 10 minutes. By contrast, we found that both *phf-10* alleles had reduced AFT (Fig. 1C), in agreement with our previous *phf-10* RNAi results (Mathies et al. 2015). The two different SWI/SNF complexes have distinct functions in the acute behavioral response to ethanol: PBAF is required for the development of AFT, while BAF is required for normal initial sensitivity to ethanol (Mathies et al. 2015). Our observation that two *phf-10* loss of function alleles have the PBAF-like phenotype of reduced AFT provides support for the idea that PHF-10 is a subunit of the PBAF complex.

## Methods

**CRISPR/Cas9 genome editing:** We chose two guide RNAs to target Cas9 to distinct sites spanning the *phf-10* gene (Fig. 1A). sgRNA clones were generated by amplifying PU6::unc-119_sgRNA plasmid (Addgene plasmid 46169) using the Q5 Site-Directed Mutagenesis kit (New England Biolabs) with forward primers RA1065 and RA1066 to introduce the guide sequence and a common reverse primer RA1063 (Friedland et al. 2013). We used co-CRISPR to identify edited worms (Kim et al. 2014). Young adult N2 animals were injected with a DNA mixture containing the *dpy-10* sgRNA in pDD162 (pSS4, 50 ng/ul), both sgRNA clones (50 ng/ul), and a *dpy-10* repair oligonucleotide to create the dominant *dpy-10(cn64)* allele. F1 roller worms were placed three to a plate and allowed to self-fertilize. Once the food was depleted, a portion of the population was washed off the plate and treated with proteinase K to produce a crude DNA prep. These DNA preps were screened using primers flanking the two Cas9 cleavage sites (RA1084 and RA1085). Individual worms were isolated from populations containing a shorter PCR product to obtain homozygous *phf-10(rd22)* mutants. The *rd21* mutation was isolated by selecting an F2 worm with a missing gonadal arm. The *phf-10* locus was amplified and sequenced to determine the molecular nature of the alleles. The mutations were backcrossed nine times, alternately to N2 and a balancer chromosome (nT1 or eT1), to remove any off-target mutations that may have been introduced during the genome editing. *phf-10(rd21)* introduces a restriction length polymorphism and was detected using PCR with primers RA1067 and RA1068, followed by *MfeI* digestion. *phf-10(rd22)* was detected using PCR with primers RA1084 and RA1085. The resulting outcrossed strains are RA588 and RA598. Primers are listed in reagents.

**Locomotion assays:** Locomotion tracking and analysis was performed as described previously (Davies et al. 2015). Briefly, assay plates were prepared by melting four copper rings into the surface of unseeded NGM plates to generate “corrals.” 100% ethanol was added to the plates at a final concentration of 0 mM or 400 mM, the plates were parafilmed, and allowed to equilibrate for one hour. Well-fed and uncrowded first‑day adult animals were transferred to a plate without food and allowed to acclimate for 30 minutes. Ten adults were transferred to each ring of an assay plate containing either 0 mM or 400 mM ethanol. After 10 and 30 minutes, 2-minute videos (1 frame per second) were taken using an Olympus SZX7 microscope with a Retiga 2000R camera (QImaging). An average speed was calculated for each ring and relative speeds (treated speed/untreated speed × 100) were used for statistical comparisons. Six trials were performed. Paired two-tailed Student’s t tests were performed in Prism 5, version 5.0a (GraphPad).

**Developmental phenotypes:** Developmental defects were assessed using a dissecting microscope. Three young adult worms were placed on a plate and allowed to lay eggs for 18 hours before removing the adults from the plate; all homozygous mutant progeny from this collection were scored. Embryonic and larval lethality was scored 24-30 hours after the end of the collection window. Gonadogenesis defects were assessed in animals that survived to the fourth larval stage. Abnormalities in the morphology of the somatic gonad, such as missing gonadal arms, were recorded. *phf-10(rd22)* homozygotes were identified in the progeny of *phf-10(rd22)/eT1[qIs60]* worms by the absence of GFP expression in the pharynx using a fluorescent dissecting microscope.

## Reagents

**Table:** 

**Strain**	**Genotype**	**Source**
N2	*C. elegans* wild isolate	CGC
JK2958	*dpy-11(e224) unc-42(e270) V/ nT1[qIs51] (IV;V)*	CGC
JK2992	*dpy-11(e224) snb-1(md247) V / eT1[qIs60] (III;V)*	(Crittenden et al. 2019)
RA588	*phf-10(rd22) V /eT1[qIs60] (III;V)*	this work
RA598	*phf-10(rd21) V*	this work

**Table:** 

**Primer**	**Name**	**Sequence**
RA1065	phf-10_sgRNA1	GGCGGCTCGGATCAATTGGgttttagagctagaaatagcaagt
RA1066	phf-10_sgRNA2	TCTCGCGTCGCGATGAAATgttttagagctagaaatagcaagt
RA1063	sgRNA_rev	CAAACATTTAGATTTGCAATTC
RA1067	phf-10_3'_for	CACGGGTTTCTAATTTCCCCC
RA1068	phf-10_3'_rev	CCGATCACATCGATCGCAGA
RA1084	phf-10_out_for	GCATAAGAGCCAGCATTGGA
RA1085	phf-10_out_rev	CACCCTCGATACTTACAATAGG
